# Hemodynamic Evaluation of Coronary Artery Lesions after Kawasaki Disease: Comparison of Fractional Flow Reserve during Cardiac Catheterization with Myocardial Flow Reserve during ^13^N-Ammonia PET

**DOI:** 10.3390/jcdd11080229

**Published:** 2024-07-23

**Authors:** Makoto Watanabe, Ryuji Fukazawa, Tomonari Kiriyama, Shogo Imai, Ryosuke Matsui, Kanae Shimada, Yoshiaki Hashimoto, Koji Hashimoto, Masanori Abe, Mitsuhiro Kamisago, Yasuhiko Itoh

**Affiliations:** 1 Department of Pediatrics, Nippon Medical School, Tokyo 113-8603, Japan; 2 Department of Radiology, Nippon Medical School, Tokyo 113-8603, Japan

**Keywords:** coronary artery lesions, myocardial flow reserve, 13N-ammonia PET, coronary fractional flow reserve, collateral vessels, Kawasaki disease

## Abstract

Coronary artery lesions (CALs) after Kawasaki disease present complex coronary hemodynamics. We investigated the relationship between coronary fractional flow reserve (FFR), myocardial flow reserve (MFR), and myocardial blood flow volume fraction (MBF) and their clinical usefulness in CALs after Kawasaki disease. Nineteen patients (18 men, 1 woman) who underwent cardiac catheterization and ^13^N-ammonia positron emission tomography, with 24 coronary artery branches, were included. Five branches had inconsistent FFR and MFR values, two had normal FFR but abnormal MFR, and three had abnormal FFR and normal MFR. The abnormal MFR group had significantly higher MBF at rest than the normal group (0.86 ± 0.13 vs. 1.08 ± 0.09, *p* = 0.001). The abnormal FFR group had significantly lower MBF at adenosine loading than the normal group (2.23 ± 0.23 vs. 1.88 ± 0.29, *p* = 0.021). The three branches with abnormal FFR only had stenotic lesions, but the MFR may have been normal because blood was supplied by collateral vessels. Combining FFR, MFR, and MBF will enable a more accurate assessment of peripheral coronary circulation and stenotic lesions in CALs and help determine treatment strategy and timing of intervention.

## 1. Introduction

In the acute phase of Kawasaki disease, the normal vascular structure, including vascular support tissues such as internal and external elastic plates, is destroyed owing to severe vasculitis, and the coronary arteries that cannot withstand the intravascular pressure dilate, resulting in coronary artery aneurysms. After the acute phase of Kawasaki disease, the dilated coronary artery undergoes active vascular remodeling for a long period, with the proliferation of the intima, narrowing of the lumen, and sometimes re-dilation [1,2]. As a result, coronary artery lesions (CALs) after Kawasaki disease are a mixture of dilated and stenotic lesions, with slow progression of the stenotic lesions, during which collateral vessels become visible in the perfusion zone of the stenotic lesions [3]. CALs often appear in multiple branches, and it is not uncommon for CALs to have complex hemodynamics. Coronary angiographic morphology is insufficient to evaluate such complex CALs, and hemodynamic evaluation is necessary for subsequent treatment.

Coronary fractional flow reserve (FFR) and coronary flow reserve (CFR) are widely used in cardiology to evaluate coronary hemodynamics and can be used to diagnose myocardial ischemia and assess its severity. The FFR has been used as an indicator for coronary revascularization, as the DEFER and FAME studies have demonstrated its validity as a criterion for determining the indication for coronary revascularization therapy [4,5]. The CFR is an index that considers the morphological stenosis of coronary arteries and the microcirculation, which is a functional abnormality [6]; it is considered an indicator of peripheral circulation and prognosis.

The agent ^13^N-ammonia PET, which has been covered by insurance in Japan since April 2012, is thought to be capable of accurately evaluating myocardial blood flow with lower radiation dose, higher sensitivity to γ-rays, and higher spatial resolution than single-photon emission computed tomography (PET) [7,8]. Therefore, it can evaluate myocardial blood flow (MBF) and myocardial flow reserve (MFR) in detail without cardiac catheterization and is considered useful in evaluating myocardial ischemia. The noninvasive evaluation of MFR with ^13^N-ammonia is generally considered to have the same clinical significance as the invasive evaluation of CFR [9].

No report has evaluated the relationship between FFR and MFR in CALs after Kawasaki disease, nor has any report evaluated myocardial blood flow by MBF. Therefore, this study retrospectively evaluated FFR, MFR, and MBF in the complex hemodynamic situation of CALs after Kawasaki disease and examined the relationship between FFR, MFR, and MBF and their usefulness in CALs after Kawasaki disease.

## 2. Materials and Methods

### 2.1. Participants

Of 32 patients with CALs after Kawasaki disease followed up at Nippon Medical School Hospital, 19 patients (18 men and 1 woman) underwent coronary angiography and FFR evaluation by cardiac catheterization and MFR evaluation by 13N-ammonia PET from April 2012 to December 2021. The patients were included in the present study. In addition, 24 coronary branches in which both FFR and MFR could be evaluated were included, because FFR was not performed in branches that did not have clear aneurysms or stenotic lesions and no lesions had been previously noted. The 24 branches included 7 right coronary arteries (RCAs), 11 left anterior descending arteries (LADs), and 6 left circumflex arteries (LCXs). The average age at examination was 17.46 years (range: 12.67–28.58 years), and the average time from onset of Kawasaki disease to examination was 14.09 years (range: 3.42–26.92 years).

All participants provided written informed consent before the examinations. The study was conducted in accordance with the Declaration of Helsinki, and the protocol was approved by the ethics committee of the Nippon Medical School (No. B-2021-360).

### 2.2. Methods

#### 2.2.1. Cardiac Catheterization

##### Coronary Angiography

Coronary angiography was used to evaluate coronary aneurysms (location, shape, and size), stenotic lesions (location and extent), and the presence of collateral vessels. Coronary aneurysms were classified as small (4–6 mm), medium (6–8 mm), and giant (>8 mm) [1]. Regression was defined as the presence of a dilated lesion in the acute phase that disappeared during the disease course and was normalized on coronary angiography. In the present study, stenotic lesions were defined as significant stenosis of ≥70% in non-left main trunk and ≥50% in left main trunk on angiography in accordance with the 2021 ACC/AHA/SCAI Guideline [10]. Based on the above definitions, the morphology of CALs was classified into regression, small aneurysm, moderate aneurysm, giant aneurysm, and stenotic complications.

##### Coronary Fractional Flow Reserve (FFR)

A 5 F or 6 F guiding catheter was inserted into the coronary artery, and a pressure wire (PressureWire^TM^ by RADI Medical Systems, Wilmington, MA, USA) was inserted into the distal part of the CAL. The intracoronary pressure (Pd) at the distal part of the CAL and the pressure at the coronary artery inlet (Pa) were measured simultaneously. The right atrial pressure (Pv) was simultaneously measured with a 5 F or 6 F balloon catheter inserted into the right atrium. FFR was calculated by measuring Pd, Pa, and Pv during maximal coronary artery filling with intracoronary papaverine hydrochloride (0.1–0.2 mg/kg). FFR was calculated using the following formula [11]:

FFR = (Pd − Pv)/(Pa − Pv)

A significant decrease in the value of FFR suggests ischemia in the perfused myocardial region of that coronary artery and is an indicator of the functional severity of regional coronary artery stenosis [5]. The reference value for children is <0.80, as with adults [12]. FFR ≥ 0.8 is considered normal, whereas FFR < 0.8 is considered abnormal.

### 2.3. ^13^N-Ammonia PET

**Figure 1:**
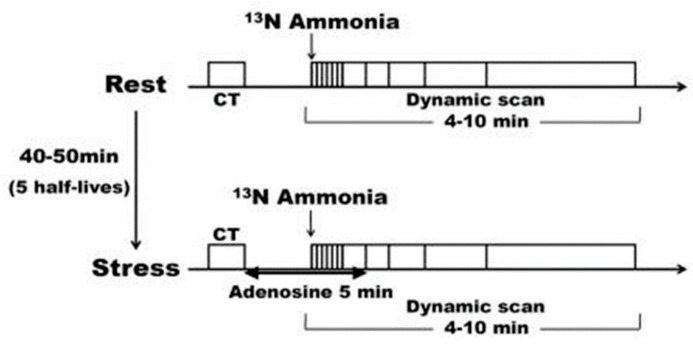
Adenosine-loaded 13N-ammonia PET examination protocol. The same protocol was used for both the resting and loading examinations except for the adenosine administration, and the interval between the resting and loading examinations was 4–5 half-lives. CT: computed tomography, PET: positron emission tomography.

The protocol for the adenosine-stressed ^13^N-ammonia PET scan is shown in Figure 1. After attenuation correction with a chest CT scan, a bolus of 7.4 MBq/kg of 13N-ammonia tracer with 30 mL of saline was administered via the right antecubital vein within 20 s, and data collection for 10 min began simultaneously with administration. Absolute MBF and MFR values were calculated by a 1-issue (intravascular—intramyocardial) 2-compartment model analysis using a list mode data from 4 min (6 s/frame × 20, 30 s/frame × 2, 60 s/frame × 1) after administration. An electrocardiogram-gated left ventricular function analysis was also performed. Pharmacological stress imaging using adenosine was initiated 40–50 min after resting imaging (4–5 half-lives). Adenosine (144 μg/kg/min) was administered through the left antecubital vein for over 5 min while monitoring the electrocardiogram, blood pressure, and oxygen saturation. Three minutes after the start of adenosine administration, a ^13^N-ammonia tracer was administered using the same tracer dose and procedure as at rest. MBF at rest and during adenosine stress, and MFR, the MBF ratio between stress and resting states, were calculated.

MFR = stress MBF/resting MBF

In the normal myocardium, vasodilators decrease vascular resistance and increase MFR. However, when coronary artery damage occurs, the increase in blood flow in the area is restricted, and the MFR is low. Myocardial ischemia occurs when MFR is <2.0 [13]; hence, MFR ≥ 2.0 is considered normal and MFR < 2.0 is abnormal.

### 2.4. Evaluation Items

Based on the above data, we compared MBF (at rest and during stress) between normal and abnormal FFR groups, compared MBF (at rest and during stress) between normal and abnormal MFR groups, compared FFR and MFR with and without collateral vessels, and examined the relationship between collateral vessels and MBF (at rest and during stress). The hemodynamics of cases with inconsistent FFR and MFR were also discussed.

### 2.5. Statistics

For continuous variables of FFR, MFR, and MBF, the data are expressed as mean ± standard deviation, and analysis of variance was used for comparisons. Fisher’s two-tailed test was used for comparison of definition scales. Statistical analysis was performed using JMP statistical software version 16 (SAS Institute Inc., Cary, NC, USA). *p* < 0.05 was considered statistically significant.

## 3. Results

There were no complications during cardiac catheterization or 13N-ammonia PET, and no adverse effects from papaverine hydrochloride or adenosine. The contrast findings of the 24 coronary branches showed two small aneurysms, seven moderate aneurysms, and nine giant aneurysms, including six cases of regression (Table 1). Six cases were complicated by stenosis (four giant aneurysms and two regressions). Six cases had an abnormal FFR < 0.8, and five had an abnormal MFR < 2.0. Sixteen patients (Cases A–P) had normal FFR and MFR, three (Cases Q–S) had abnormal values for both, three (Cases V–X) had abnormal FFR, and two (Cases T and U) had abnormal MFR. All six patients with stenosis had abnormal FFR, and three of them had collaterals. In addition, the collateral blood circulation in case T was supplying the LAD and RCA regions from the LCX and not the receiving side of the LCX region. Six cases underwent coronary artery bypass grafting (CABG) after the examination.

**Figure 2:**
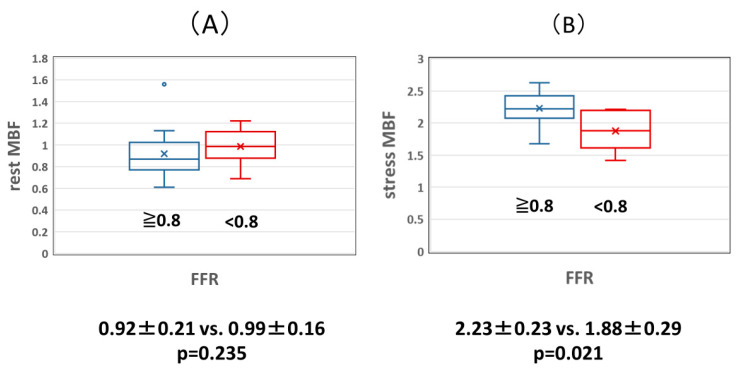
(**A**) Comparison of resting MBF in the normal and abnormal FFR groups. There was no significant difference in resting MBF between the normal and abnormal FFR groups. (**B**) Comparison of stress MBF in the normal and abnormal FFR groups. The abnormal FFR group had significantly lower stress MBF than the normal FFR group. FFR: coronary fractional flow reserve, MBF: myocardial blood flow volume fraction.

**Figure 3:**
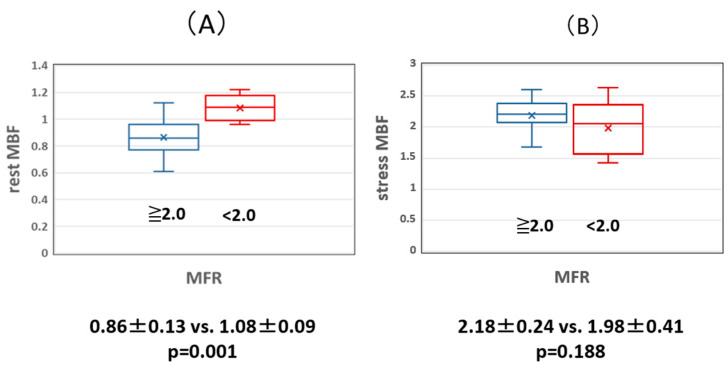
(**A**) Comparison of resting MBF in the normal and abnormal MFR groups. The rest MBF was significantly higher in the abnormal MFR group than in the normal MFR group. (**B**) Comparison of stress MBF in the normal and abnormal MFR groups. There was no significant difference in stress MBF between the normal and abnormal MFR groups. MBF: myocardial blood flow volume fraction, MFR: myocardial flow reserve.

The relationship between MBF, FFR, and MFR was examined. Although there was no significant difference in resting MBF between the normal and abnormal FFR groups (0.92 ± 0.21 vs. 0.99 ± 0.16, *p* = 0.235), the MBF during adenosine loading was significantly lower in the abnormal FFR group than in the normal FFR group (2.23 ± 0.23 vs. 1.88 ± 0.29, *p* = 0.021) (Figure 2). MBF at rest in the abnormal MFR group was significantly higher than that in the normal MFR group (0.86 ± 0.13 vs. 1.08 ± 0.09, *p* = 0.001), but MBF during adenosine loading was not significantly different between the normal and abnormal MFR groups (2.18 ± 0.24 vs. 1.98 ± 0.41, *p* = 0.188) (Figure 3).

In the abnormal FFR group with stenosis in all cases, rest and stress MBF were examined in the presence and absence of collateral vessels, respectively. Resting MBF was significantly lower with collateral vessels (0.86 ± 0.12 vs. 1.11 ± 0.08, *p* = 0.041). Moreover, stress MBF did not differ significantly between patients with and without collateral vessels (2.03 ± 0.25 vs. 1.73 ± 0.26, *p* = 0.149) but tended to be higher when collateral vessels were present. Therefore, MFR was significantly higher in the presence of collateral blood vessels (Table 2). Regarding classification by aneurysm size, no stenosis, abnormal FFR, abnormal MFR, or collateral vessels were observed in small and medium aneurysms.

Among the cases with inconsistent FFR and MFR results, ^13^N-ammonia PET images and coronary angiography images of Cases T, U, and V are shown.

**Figure 4:**
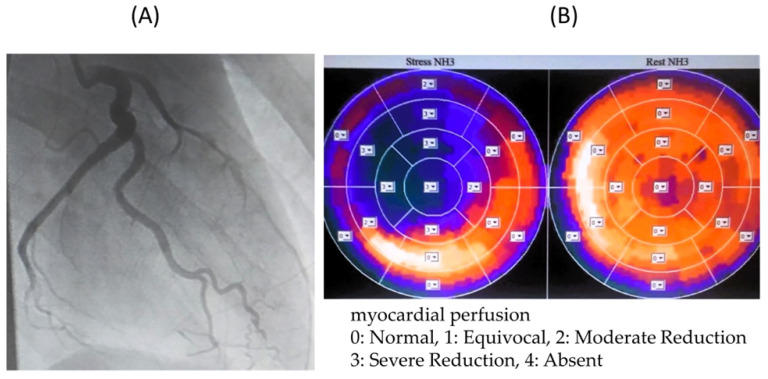
(**A**) Coronary angiography image of Case T. (**B**) 13N-ammonia PET image of Case T. Owing to occlusion of the LAD and RCA, only LCX is responsible for coronary circulation. MFR may be low due to high blood flow at rest. LAD: left anterior descending artery, LCX: left circumflex artery, MFR: myocardial blood flow reserve, RCA: right coronary artery, PET: positron emission tomography.

In Case T (Figure 4), the LAD and RCA were occluded, and the LCX alone was responsible for the coronary circulation, with collateral blood flow from the LCX to the LAD and RCA regions. Therefore, MFR was thought to be low because of the high resting blood flow of 1.56 mL/min/g in the LCX. We judged that there was no myocardial ischemia in the perfused area of the LCX, and no therapeutic intervention was performed on the LCX.

**Figure 5:**
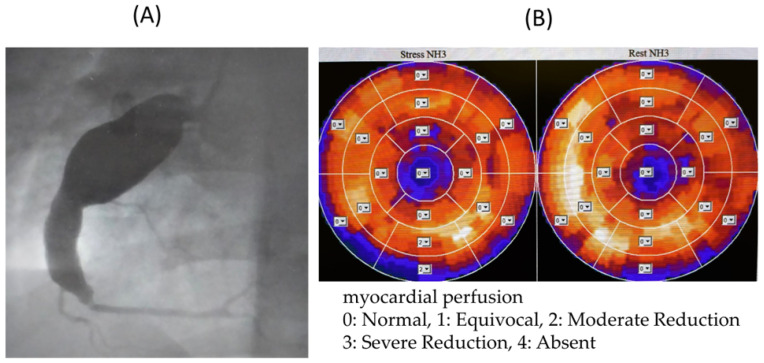
(**A**) Coronary angiography image of Case U. (**B**) 13N-ammonia PET image of Case U. The FFR was normal because of the giant aneurysm, and the MFR was low because the peripheral coronary arteries were already dilated at rest. FFR: coronary fractional flow reserve, MFR: myocardial flow reserve, PET: positron emission tomography.

In Case U (Figure 5), the FFR was normal because the aneurysm was so large that a pressure gradient was unlikely to occur, and the MFR was low because the MBF at rest was as high as 1.13 mL/min/g because the peripheral coronary arteries were already dilated at rest.

**Figure 6:**
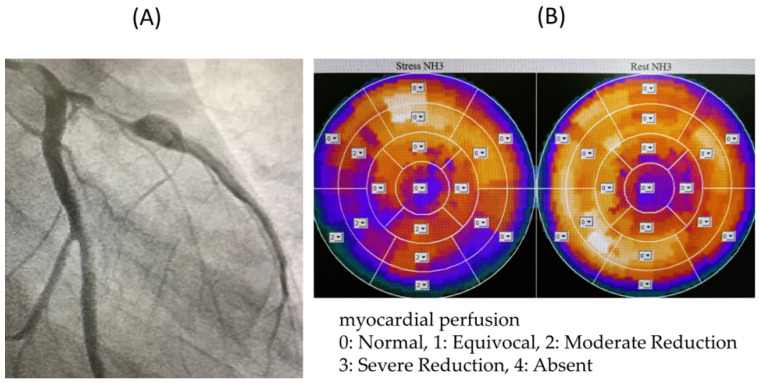
(**A**) Coronary angiography image of Case V. (**B**) 13N-ammonia PET image of Case V. The FFR was low due to the presence of a stenotic lesion, but it was thought the peripheral circulation was not impaired due to the collateral blood vessels. FFR: coronary fractional flow reserve; PET: positron emission tomography.

In Cases V (Figure 6), W, and X, only the FFR was abnormal, and although there was no evidence of myocardial peripheral circulatory disturbance, the FFR was thought to be low owing to a stenotic lesion. All three patients were considered to have significant stenotic lesions and underwent CABG.

## 4. Discussion

In our study, FFR and MFR are indicators of myocardial ischemia, but even in CALs after Kawasaki disease, FFR is abnormal in all cases of stenosis, and stress MBF was also significantly lower in the group with abnormal FFR, which may be a useful indicator of stenotic lesions. On the other hand, ^13^N-ammonia PET can calculate MFR, an index that takes peripheral circulatory disturbance into account, as well as the absolute value of myocardial blood flow at rest and under load. Although the presence of collateral vessels and high resting coronary blood flow can result in low MFR values even in the absence of peripheral circulatory impairment, more detailed assessment of coronary circulatory dynamics is possible by considering the absolute value of MBF.

FFR is a measure of the percentage contribution of vascular regional lesions to overall blood flow impairment, and the DEFER and FAME studies have shown the validity of FFR as a criterion (FFR < 0.8) to determine the indication for revascularization therapy [4,5]. Conversely, CFR is reduced owing to increased resting coronary blood flow and decreased blood flow during maximal coronary dilatation. Resting coronary blood flow increases with increasing heart rate, systolic blood pressure, and myocardial contractility. Resting coronary blood flow also increases when epicardial stenosis is present because the microvasculature dilates from rest to increase the vascular bed and decrease peripheral vascular resistance to prevent the myocardium from becoming ischemic. Maximal coronary blood flow at the diastole is reduced by local vascular lesions and myocardial microcirculatory disturbances. In other words, CFR is an index that considers the morphological stenosis of the coronary arteries and the functional abnormality of the microcirculation [6]. Cases with CFR ≥ 2.0 have a cardiovascular accident rate of <1.0%, and CFR is considered an indicator of peripheral circulation and prognosis [14].

Inconsistent cases between anatomic stenosis and CFR have been reported in adult cardiology, and the discrepancy between CFR and FFR may reflect the extent and scope of focal and diffuse epicardial coronary artery disease and the presence of microvascular dysfunction [15]. Considering the relationship between both indicators, it is inferred that cases with an FFR of ≥0.8 and a CFR of <2.0 are those with a strong microcirculatory disturbance, whereas cases with an FFR of ≤0.8 and a CFR of ≥2.0 are local lesions with adequate microcirculation [16]. The prognosis of patients with CFR < 2.0 and FFR > 0.8 was reported to be the worst, whereas that of patients with CFR > 2 and FFR < 0.8 was almost equal to that of the group with both indices at normal values [6].

In this study, five branches had inconsistent FFR and MFR results. Two branches (Cases T and U) with abnormal MFR only had no stenotic lesions and normal FFR. In Case T, the LCX alone was responsible for the coronary circulation, and the MFR was thought to be low because of the high resting MBF. Case U showed no obvious stenotic lesions, but because of the giant aneurysm, the blood flow velocity was reduced, and it was assumed that the small coronary artery was already highly dilated at rest. In general, peripheral vascular resistance decreases with the progression of stenosis in coronary artery lesions; the more advanced the stenosis, the more dilated the small coronary arteries become to reduce resistance and maintain constant myocardial blood flow at rest. This decreases vasodilatory reserve and reduces stress MBF and CFR [17]. However, since Cases U and T were children or young adults, even if resting MBF was elevated, they still had sufficient remaining coronary vasodilatory reserve; both stress MBF values were >2.0, suggesting that these patients did not have myocardial ischemia. It has been reported that even in cases with coronary aneurysms, low MFR is associated with a depressed coronary vascular resistance response [18]; the present study showed that resting MBF was significantly higher in the group with abnormal MFR, suggesting that the peripheral coronary arteries were dilated at rest. The fact that the stress MBF did not differ significantly between the normal and abnormal MFR groups suggests that children and young adults maintain a high vasodilatory reserve, unlike middle-aged and older patients with epicardial stenosis who have clinical risk factors and advanced atherosclerosis, including microangiopathy.

The six branches with abnormal FFR all had more than 75% stenotic lesions on contrast. As stenotic lesions in coronary arteries progress, the CFR decreases, and its value correlates with the stenosis rate [17]. For the six branches with abnormal FFR only, the reason for the normal MFR values besides the negative presence of peripheral circulatory disturbance and despite stenosis of >90% in three branches was speculated to be the presence of collateral vessels. Although quantification of MBF and MFR by PET has been evaluated as an accurate tool to detect obstructive coronary artery disease and has been reported to correlate well [19,20,21], there are no reports on CALs after Kawasaki disease with complex hemodynamics, including collateral vessels. We also examined collateral vessels in relation to FFR, MFR, and MBF and found that resting MBF was significantly lower and stress MBF tended to be higher when collateral vessels were present. Therefore, MFR was normal in cases with collateral vessels. This is thought to be due to the low myocardial blood flow, which led to the presence of collateral vessels, while the presence of collateral vessels contributed to the increased myocardial blood flow during adenosine loading. In other words, all three branches with collateral vessels received blood supply from collateral vessels in the dominant region. Thus, MBF was maintained under load, which may have resulted in normal MFR values.

As mentioned above, a high blood flow (stress MBF) at maximal coronary dilatation increases the pressure gradient before and after a stenotic lesion, decreasing FFR. The LAD has a large perfusion area, and the increase in blood flow during maximal coronary dilatation is considered significant. Therefore, a large increase in blood flow through the stenotic lesion increases the pressure gradient and decreases FFR [6]. In this case, FFR would be treated as a false positive, but since the three branches in this study have stenotic lesions, the abnormal FFR value was considered significant.

Since stress MBF can be high even when FFR and CFR are abnormal, it seemed important to consider MBF, along with FFR and MFR, in addition to morphological diagnosis including collateral vessels. As a result, more detailed assessment of peripheral circulation and stenotic lesions in the coronary arteries may be useful in determining treatment strategy and timing of intervention. In this study, stress MBF tended to be high, and if the stress MBF is high enough, ischemia during exercise is unlikely to occur. Even if FFR and MFR are low, surgical intervention may not be required if sufficient stress MBF is maintained, so further findings are needed.

The study had some limitations. The number of cases is limited, and the absolute number is small. In addition, low FFR values and the stenotic lesions on angiography were consistent in all cases, but especially since the number of cases was only six, further accumulation of cases is needed. Although cardiac function may be impaired in cases of suspected myocardial peripheral circulatory disturbance, we could not assess cardiac function in cases with low MFR. Furthermore, only the presence or absence of collateral vessels was evaluated in this study. The blood flow in the collateral vessels was not evaluated, necessitating a more detailed evaluation of collateral blood flow involvement in the future.

## 5. Conclusions

Evaluation of myocardial ischemia by CALs was studied using FFR from cardiac catheterization and MFR and MBF from ^13^N-ammonia PET. FFR is useful in evaluating stenotic lesions, but if collateral vessels are well developed, stress MBF may be high and may not require surgical intervention.

## Figures and Tables

**Table 1:** Case background.

Case	Site	AN	Stenosis	FFR	MFR	Resting MBF	Stress MBF	Collateral	Intervention	Stenosis Ratio (%)
A	#8	reg	-	0.93	2.61	0.96	2.6	-	-	
B	#2	giant	-	0.82	2.3	0.9	2.07	-	-	
C	#13	mod	-	0.91	2.28	1.06	2.42	-	-	
D	#7	mod	-	0.96	2.89	0.77	2.24	-	-	
E	#7	giant	-	0.8	2.76	0.61	1.68	-	-	
F	#12	mod	-	0.93	2.22	1.10	2.26	-	-	
G	#2	giant	-	0.83	3.26	0.77	2.5	-	-	
H	#2	small	-	0.96	2.19	1.12	2.45	-	-	
I	#7	mod	-	0.95	2.75	0.8	2.21	-	-	
J	#1	small	-	0.88	3.18	0.75	2.38	-	-	
K	#7	giant	-	0.83	2.48	0.86	2.13	-	-	
L	#2	mod	-	0.95	2.34	0.85	1.99	-	-	
M	#9	mod	-	0.95	2.14	0.99	2.11	-	-	
N	#13	mod	-	0.85	2.43	0.88	2.14	-	-	
O	#4	reg	-	0.94	2.43	0.77	2.24	-	-	
P	#13	reg	-	0.93	2.29	0.75	1.99	-	-	
Q	#7	giant	+	0.6	1.68	1.02	1.71	-	CABG	90
R	#8	reg	+	0.5	1.3	1.09	1.42	-	CABG	99
S	#7	giant	+	0.75	1.67	1.22	2.05	-	CABG	75
T	#13	reg	-	1.00	1.69	1.56	2.63	(+) *	-	
U	#2	giant	-	0.96	1.83	1.13	2.08	-	-	
V	#8	reg	+	0.44	2.33	0.94	2.19	+	CABG	99
W	#7	Giant	+	0.73	2.33	0.95	2.21	+	CABG	90
X	#11	Giant	+	0.55	2.42	0.69	1.68	+	CABG	99

AN: aneurysm, FFR: coronary fractional flow reserve, MFR: myocardial flow reserve, MBF: myocardial blood flow, reg: regression, mod: moderate, CABG: coronary artery bypass grafting. * The collateral blood vessel in Case T supplies the LAD (left anterior descending artery) and RCA (right coronary artery) regions and is not counted in the number of cases because it is not on the receiving side.

**Table 2:** Comparison of FFR, MFR, resting MBF, and stress MBF with and without collateral blood flow.

	FFR	MFR	Resting MBF	Stress MBF
Collateral (+)	0.57 ± 0.12	2.36 ± 0.04	0.86 ± 0.12	2.03 ± 0.25
Collateral (-)	0.62 ± 0.10	1.55 ± 0.18	1.11 ± 0.08	1.73 ± 0.26
*p*-value	0.359	0.009	0.041	0.149

FFR: coronary fractional flow reserve, MFR: myocardial flow reserve, MBF: myocardial blood flow.

## Data Availability

The original contributions presented in the study are included in the article material; further inquiries can be directed to the corresponding author.
